# An Efficient One-Pot Protocol for the Synthesis of Substituted 3,4-Dihydropyrimidin-2(1*H*)-ones Using Metallophthalocyanines (MPcs) as Potent Heterogeneous Catalysts: Synthesis, Characterization, Aggregation and Antimicrobial Activity

**DOI:** 10.3390/molecules22040605

**Published:** 2017-04-09

**Authors:** Naceur Hamdi, Rawdha Medyouni, Hallouma Bilel, Lamjed Mansour, Antonio Romerosa

**Affiliations:** 1 Research Laboratory of Environmental Sciences and Technologies (LR16ES09), Higher Institute of Environmental Sciences and Technology, University of Carthage, Hammam-Lif 2050, Tunisia; rawdha.medyoni@gmail.com (R.M.); bilelhalouma@gmail.com (H.B.); 2 Chemistry Department, College of Science and Arts, Qassim University, Al-Rass 51921, Saudi Arabia; 3 Zoology Department, College of Science, King Saud University, P.O. Box 2455, Riyadh 11451, Saudi Arabia; lamjed.mansour@gmail.com; 4 Área de Química Inorgánica-CIESOL, Universidad de Almería, 04120, Almería, Spain; romerosa@ual.es

**Keywords:** Biginelli reaction, dihydropyrimidinones, metallophthalocyanine derivatives, aggregation, antimicrobial activity

## Abstract

In this study, novel phthalonitrile **3** and their corresponding metal-free **4** and metallophthalocyanine derivatives **5**–**7** bearing 2-isopropenyl-4-methoxy-1-methylbenzene groups were synthesized and characterized. 3,4-Dihydropyrimidinones have been synthesized by a modified Biginelli-type reaction with various metallophthalocyanines **5**–**7** as catalysts. Compared to the classical Biginielli reaction, the new method has the advantages of good yield and short reaction time. Among the various metallophthalocyanines studied, cobalt (II)-phthalocyanine was found to be most active for this transformation. The newly prepared compounds were characterized using elemental analyses, MS, IR, ^1^H/^13^C-NMR and UV-Vis spectroscopy. In addition; the 3,4-dihydropyrimidinones (DHPMs) **8**–**12** were investigated for antimicrobial activities and revealed good activity. The minimum inhibitory concentration (MIC) was determined by the microdilution technique in Mueller-Hinton broth. The MICs were recorded after 24 hours of incubation at 37 °C. These results are promising, showing these compounds are biologically active.

## 1. Introduction

In 1893 Biginelli reported the first synthesis of dihydropyrimidines of type **8**–**12** by a simple one-pot condensation reaction of ethyl acetoacetate, benzaldehyde, and urea [1,2]. In the following decades the original cyclocondensation reaction has been extended widely to include variations in all three components, allowing access to a large number of multifunctionalized dihydropyrimidine derivatives [3]. Largely ignored for many years, the Biginelli reaction has recently attracted a great deal of renewed attention, and several improved procedures for the preparation of dihydropyrimidines of type **8**–**12** have been reported within the past few years [4,5]. Various solid phase modifications of the Biginelli reaction suitable for combinatorial chemistry have also been described [6,7,8,9]. The Biginelli reaction is a multiple-component chemical reaction that creates 3,4-dihydropyrimidin-2(1*H*)-ones from ethyl acetoacetate, an aryl aldehyde (such as benzaldehyde ), and urea [10].The reaction can be catalyzed by Brønsted acids and/or by Lewis acids such as copper(II) trifluoroacetate hydrate and boron trifluoride. Furthemore running this reaction under heterogeneous condition is more promising since it involves the facile recovery and reuse of the expensive catalyst [11,12,13,14,15,16,17,18,19,20].

**Scheme 1:**
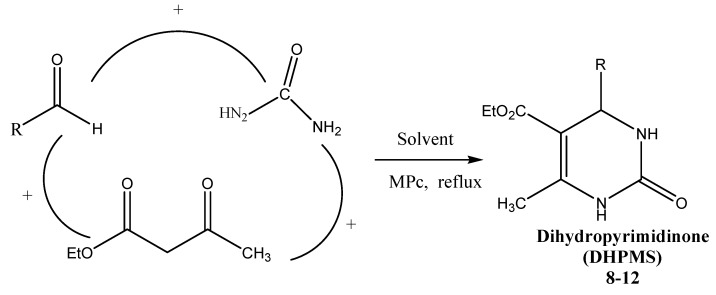
General one-pot Biginelli reaction to generate 3,4-dihydropyrimidinones **8**–**12**.

In the current work, we report an efficient procedure for the synthesis of 3,4-dihydropyrimidin-2(1*H*)-ones through one pot cyclocondensation of aldehyde, urea and ethylacetate compounds using metallophthalocyanines as reusable heterogeneous catalysts. Additionally their antibacterial activities against Gram-positive as well as gram-negative bacteria followed by MIC determination were evaluated (Scheme 1). 

## 2. Results

**Scheme 2:**
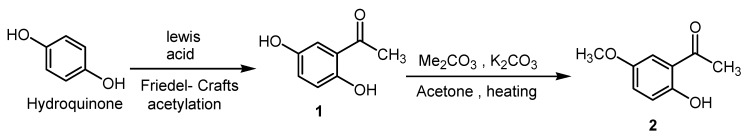
Regioselective methylation of quinacetophenone **1**.

Acetylation of hydroquinone can be achieved by treating hydroquinone with an acid chloride or anhydride in the presence of a Lewis acid. Rosenmund and Lohfert reported the synthesis of quinacetophenone **1** through the reaction of hydroquinone with acetyl chloride in the presence of aluminum chloride under thermal conditions [21]. The following text describes the synthesis of the phthalonitrile ligand **3**, and its applications. The non-hydrogen bonded, less crowded hydroxyl group of quinacetophenone **1** can be methylated regioselectively by the reaction with Me_2_CO_3_ in the presence of potassium carbonate yielding 2-hydroxy-5-methoxyacetophenone **2** in moderate yield (Scheme 2).

Spectral data were coherent with the proposed structure **2**. According to the IR spectral results of the compound **2**, characteristic OH stretching vibration was observed at 3315 cm^−1^. In the ^1^H-NMR spectrum of **2**, the disappearance of one OH peak of **1** and the presence of additional methylic protons at δ 2.2 ppm indicated that the synthesis of compound **2** was accomplished. The signals of aromatic protons were observed at δ 7.33–7.85 ppm. In ^13^C-NMR of **2**, the new peak at 50.8 ppm and 195.6 ppm belonging to OCH_3_ and the (CO) lactone carbons indicated that the acylation has occurred. Mass spectrum of compound **2** indicated that target compound was successfully prepared. Also elemental analysis data of compound **3** was satisfactory.

**Scheme 3:**
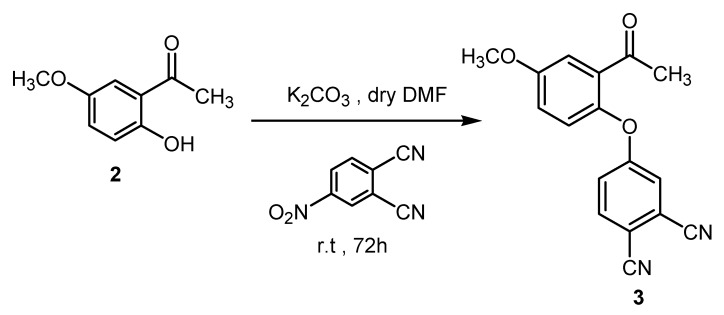
Synthesis of compound **3**.

The procedure for the synthesis of compound **3** was similar to that used for many examples in the literature [22,23]. Phthalonitrile derivative **3** was prepared from 4-nitrophthalonitrile by nucleophilic substitution of the nitro group with the OH function of compound **2** via SNAr reaction in polar aprotic dry solvents (DMSO or DMF) [24,25] in the presence of potassium carbonate as basic catalyst K_2_CO_3_ with 90% yield (Scheme 3).

The structures of new compound **3** were confirmed by the combination of IR, ^1^H-NMR, ^13^C-NMR and elemental analysis. Spectral investigations of the dinitrile derivative show good agreement with proposed structure of compound **3**. The IR spectrum of compound **3** had a strong acetyl C=O vibration absorption at about 1720 cm^−1^ and displayed absorption at about 2230 cm^−1^ assigned to CN function. Furthermore, the formation of compound **3** was obviously verified by the disappearance of the OH vibration of compound **2** and NO_2_ vibration of 4-nitrophthalonitrile. In the ^1^H-NMR spectrum of **3**, the OH group of compound **2** disappeared, as expected, and the appearance of new signals confirmed the proposed structure. The signals of aromatic protons were observed at δ 7.36–8.26 ppm.^13^C-NMR spectral data of **3** show significant peaks for nitriles, OCH_3_ and COCH_3_ carbon atoms at δ 116.07 ppm, 113.25 ppm, 57.8 ppm and 175.5 ppm respectively.

**Scheme 4:**
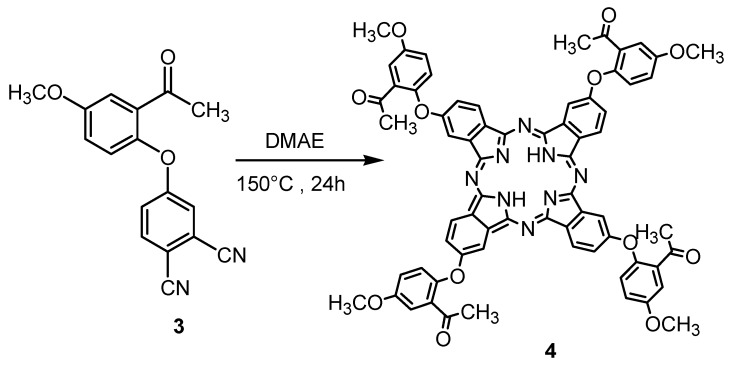
Synthesis of unmetalled phthalocyanine compound **4**.

The self-condensation of the dicyanobenzene compound **3** in a high-boiling solvent 2-(dimethylamino) ethanol (DMAE) in the presence of a few drops 1,8-diazabicyclo[5.4.0]undec-7-ene (DBU) at 150 °C under a nitrogen atmosphere for 24 h afforded unmetalled phthalocyanine compound **4** with moderate yield after purification by column chromatography method utilizing chloroform/methanol (91:9) as eluent (Scheme 4).

The structure of metal-free phthalocyanine compound **4** was verified by FT-IR, ^1^H-NMR and UV-Vis spectroscopic methods, as well as by elemental analysis. All the analytical spectral data are consistent with the predicted structure.

The disappearance of the CN stretching vibration on the IR spectra of phthalonitrile compound **3** suggested the formation of compound **4**. In addition in the IR spectrum of compound **4**, stretching vibrations of the acetyl C=O vibration at 1725 cm^−1^ and aromatic CH at 3056 cm^−1^ appeared at expected frequencies. In the ^1^H-NMR spectrum of compound **4**, the aromatic protons appeared at δ: 7.05–8.55 ppm, the aliphatic CH_3_ protons appeared at 2.94 ppm and the methoxylic protons appeared at δ: 3.68 ppm.

**Scheme 5:**
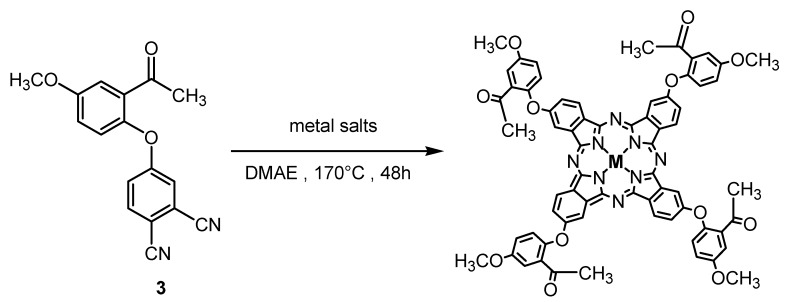
Synthesis of metallophthalocyanine derivatives **5**–**7**.

Metallophthalocyanines **5**, **6**, and **7** were obtained from the reaction of phthalonitrile derivative **3** with corresponding anhydrous metal salts CuCl_2_·2H_2_O for complex **5**, CoCl_2_·6H_2_O for complex **6**, and ZnCl_2_ for complex **7** in 2-(dimethylamino) ethanol and DBU at 170 °C for 48 h under a nitrogen atmosphere after purification by column chromatography method utilizing chloroform/methanol (80:20) as eluent (Scheme 5).

Characterization of the phthalocyanine compounds was achieved by analysis of spectroscopic data from NMR (for complexe **6**), IR, UV-vis, elemental analysis and mass spectroscopy. 

In the IR spectra of the phthalocyanines **5**–**7**, the proof of the cyclotetramerization was the absence of the CN stretching vibration observed at 2230 cm^−1^ of the compound **3**. The main differences between metal-free (compound **4**) and metallophthalocyanines **5–7** are inner cores NH stretching vibrations observed at 3406 cm^−1^ in the IR spectra, respectively.

^1^H-NMR and ^13^C-NMR spectra of the metallophthalocyanines (M: Cu, Co) were precluded due to having paramagnetic metal atom [26,27]. Elemental analysis results of all compounds show good agreement with the calculated values.

^1^H-NMR spectra of the Zn phthalocyanine derivative **6** were obtained in DMSO-*d*_6_ at room temperature. The aromatic protons were observed at δ 7.36–8.26 ppm. 

### 2.1. Ground State Electronic Absorption and Aggregation Properties 

**Figure 1:**
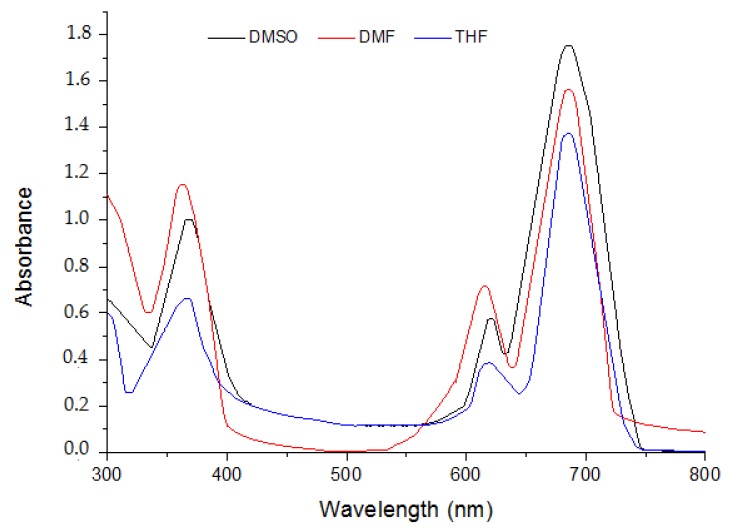
Absorption spectra of MPc **5** in different solvents at the concentration (Molar, 10^−5^ M).

**Figure 2:**
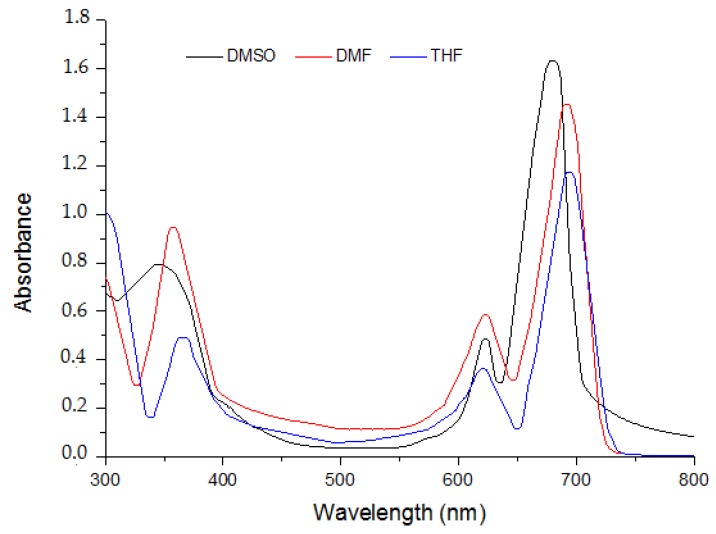
Absorption spectra of MPc **6** in different solvents at the concentration (Molar, 10^−5^ M).

**Figure 3:**
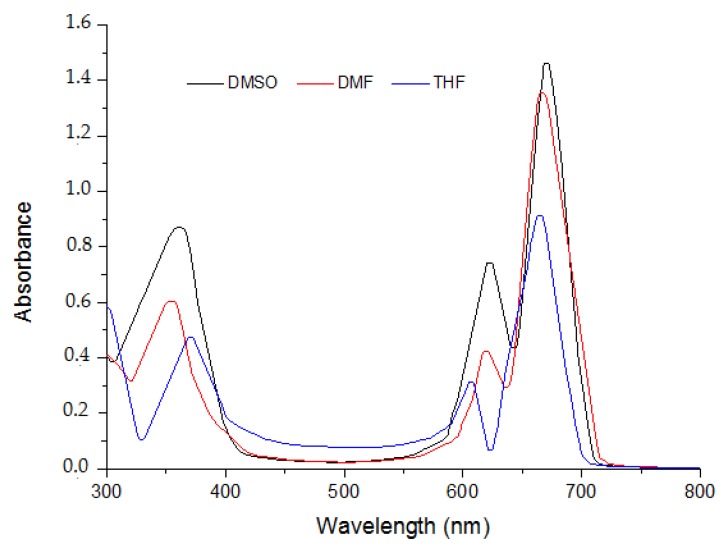
Absorption spectra of MPc **7** in different solvents at the concentration (Molar, 10^−5^ M).

The spectral profiles of the three phthalocyanine compounds **5**–**7** were recorded in three organic solvents DMF, DMSO and THF. The spectra of the phthalocyanines **5**–**7** in various organic solvents are presented in Figure 1, Figure 2 and Figure 3. 

It is obvious that there is no considerable change in absorption profile of the molecules with variation in polarity of the medium. The given compounds **5** and **6** possess bathochromic spectral shifts (positive solvatochromism) while moving from least polar solvent (DMSO) to the most polar solvent (THF). This is attributed to the fact that molecule in the ground state and excited state possesses different polarities. Absorption and extinction coefficients are presented in Table 1.

They exhibited typical electronic absorption with single intense π-π* transition, referred to as the Q band, much intense and characteristic for phthalocyanines, and another less intense and broader π-π* transition which is so-called Soret band (B).

### 2.2. Aggregations Studies 

**Figure 4:**
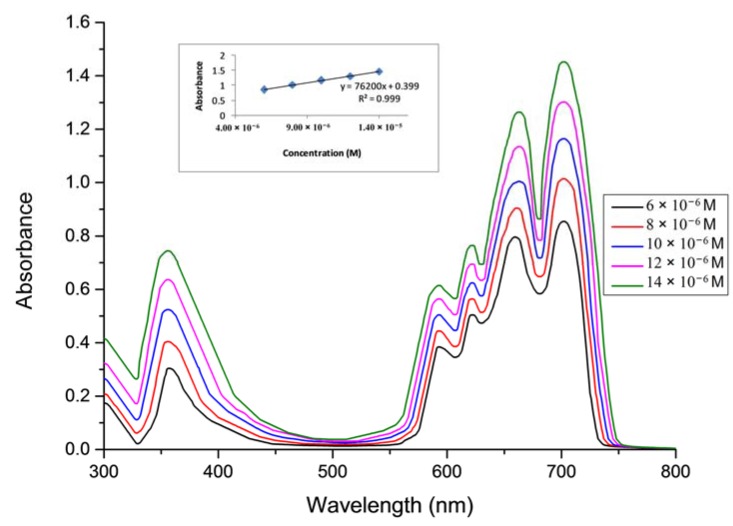
The aggregation behavior of phthalocyanine **5** in DMSO.

**Figure 5:**
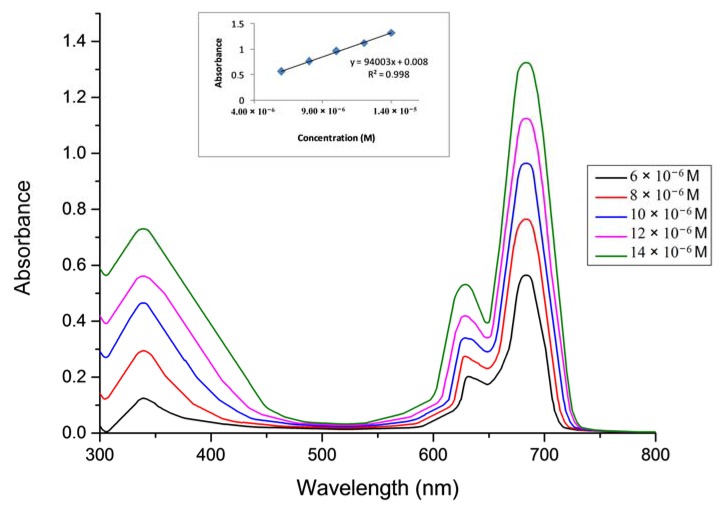
The aggregation behavior of phthalocyanine **6** in DMSO.

**Figure 6:**
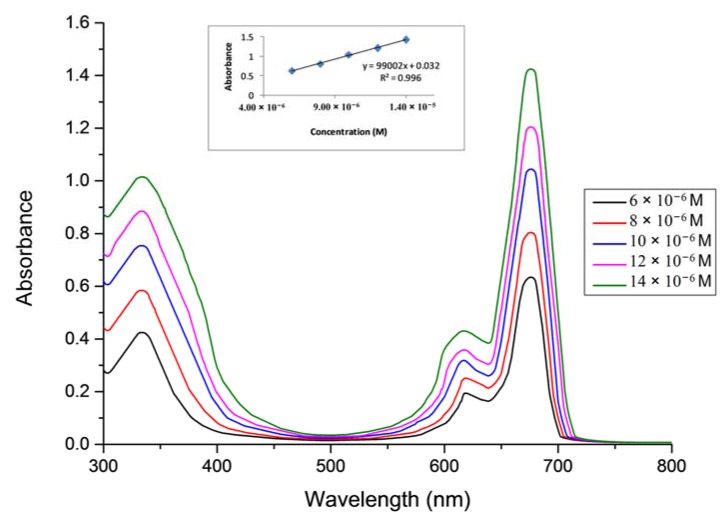
The aggregation behavior of phthalocyanine **7** in DMSO.

In this study, the aggregation behavior of complexes **5**–**7** was examined at different concentrations in DMSO [28,29,30,31]. (Figure 4 for complex **5**, Figure 5 for complex **6** and Figure 6 for complex **7**).

The aggregation behaviours of Pcs (**5**–**7**) were investigated at different concentrations ranging from 1.20 × 10^−6^ to 14 × 10^−6^ M in DMSO. It was observed that all of the Pcs (**5**–**7**) are consistent with the Beer-Lambert law for these concentration ranges.

### 2.3. Catalytic Eficiency of Metallophthalocyanines ***5**–**7***

The cyclocondensation of benzaldehyde, ethylacetoacetate and urea was studied by using phthalocyanines **5**–**7** as catalysts. These results are summarized in Table 2.

The DMC was found to be the better solvent in terms of reaction time and yield than all other solvents tested such as THF, ethanol and acetone. Furthermore a variety of different aromatic aldehydes were reacted with ethylacetoacetate and urea in the presence of a catalytic amount of Co(II)Pc **5** under similar reaction conditions. Results of these experiments are given in Table 3. 

The new compounds **8**–**12** were characterized by IR, ^1^H-NMR spectroscopies and elemental analysis. The analyses are consistent with the predicted structures as shown in the experimental section.

The data obtained from the ^1^H-NMR spectrum of compound **9** provided the characteristic chemical shifts for the structures, as expected. The present protocol provides a high yielding, efficient and improved route for the synthesis of dihydropyrimidinones. Further, Co(II)Pc **5** catalyst is better than the usual H^+^, ZnCl_2_ and ammonium salt catalysts used previously for the synthesis of (DHPMs) [32,33].

### 2.4. Recycling Performance of the Catalysts ***5**–**7***

After separation of the products, the filtrate containing the catalyst was reused in the next run without further purification. The data listed in Table 4 show that the Co(II)-phthalocyanine **5** could be reused three times without a notable decrease of catalytic activity. The easy recycling performance is also an attractive property of the Co(II)-phthalocyanine **5** for environmental protection and economic reasons.

The obtained results from Table 4 indicate that the catalyst can be reused as such without further treatment. 

### 2.5. Antimicrobial Activity 

The antimicrobial activity of the tested compounds **8**–**12** was evaluated against common pathogenic bacteria. They are are given in Table 5. Significant antimicrobial activity (MIC = 0.312 mg/mL) was observed against M. luteus. The antibiotic activity is no good (barely miliMolar) .They are slightly active. The best compound is not from a MCR.

## 3. Experimental Section

### 3.1. General Information

All solvents and commercially available reagents were purchased from the suppliers and used without further purification. ^1^H-NMR(300 MHz) and ^13^C-NMR(75 MHz) spectra were recorded using (Bruker DPX 300, University of Almeria, Spain). Spectra were recorded in DMSO solutions and chemical shifts are reported in parts per million (ppm) relative to tetramethylsilane (TMS) as the standard. NMR multiplicities are abbreviated as follows: s = singlet, d = doublet, t = triplet, m = multiplet signal. IR spectra were recorded on a 398 spectrophotometer (Perkin-Elmer, King Saud University, Ryadh, Saudi Arabia). Elemental microanalysis was performed on an Elementar Vario El III Carlo Erba 1108 elemental analyzer (INRAP, Sidi Thabet, Tunisia) and the values found were within ±0.3% of the theoretical values. 

### 3.2. Synthesis of 4-(2-Acetyl-4-methoxyphenoxy)phthalonitrile *(**3**)*

To a stirred solution of 4-nitrophthalonitrile (0.38 g, 1.92 mmol) and 1-(2-hydroxy-5-methoxy-phenyl)ethanone (0.55 g, 1.92 mmol) in anhydrous DMF (15 mL) was added portionwise finely powdered anhydrous potassium carbonate (0.8 g, 5.76 mmol) and the resulting mixture was stirred at room temperature for 24 h. The crude product was collected by filtration recrystallized from THF-petroleum ether to afford a white powder. Yield: 0.77 g (98%). m.p. = 400 °C. FT-IR (KBr) ν_max_, cm^−1^: 1305 (C-N), 1568 (C=C), 2230 (C≡N), 3049 (C-H, aromatic). ^1^H-NMR δ ppm: 7.35–8.26 (m, 6H, H_arom_), 3.60 (s, 3H, OCH_3_), 2.02 (s, 3H, CH_3_). ^13^C-NMR δ: 19.9 (CH_3_), 116.07 (CN), 113.25 (CN), 116.4–135.4 (C_arom_). MS (LCMS-MS) *m*/*z*: Calc. 292.2; found: 292.2. Anal. Calc. for C_17_H_12_O_3_N_2_: C, 69.85%; H, 4.13%; N, 9.58%; found: C, 69.8%; H, 4.1%; N, 9.4%.

### 3.3. Synthesis of Metal-Free ***4***

Compound 3 (0.38 g, 1 mmol), dry *N*,*N*-dimethylaminoethanol (DMAE) (4 mL), three drops of 1,8-diazabicyclo[5.4.0]undec-7-ene (DBU) were placed in a standard a Schlenk tube under a nitrogen atmosphere and the mixture was refluxed for 24 h at 150 °C. After cooling to room temperature, the reaction mixture was precipitated by the addition of ethanol and this green product was filtered off. The raw product was purified by silica gel column chromatography. Yield: (75%). m.p. = 330 °C. FT-IR (KBr) ν_max_, cm^−1^: 3406 (N-H); 3056 (C-H_arom_); 1232 (C-N)_arom_; 1613 (C=C). UV/Vis (DMSO, λ_max_ nm (log ε)): 340 (4.684), 605 (4.377), 624 (4.528), 658 (5.004), 697 (5.117). ^1^H-NMR δ ppm: 7.05–8.55 (m, 24H, H_arom_), 3.68 (s, 12H, OCH_3_), 2.94 (s, 12H, CH_3_). Calc. for (C_67_H_38_N_8_O_12_): calculated (C, 70.15%; H, 3.33%; N, 9.76%); found (C, 70.1%; H, 3.20%; N, 9.8%).

### 3.4. General Procedure for the Synthesis of Metallophthalocyanines ***5**–**7***

Compound 3 (0.24 mmol), 0.06 mmol of the corresponding metal salts (ZnCl_2_, CuCl_2_•2H_2_O, CoCl_2_•6H_2_O), *N,N*-dimethylaminoethanol (DMAE) (4 mL) and 1,8-diazabicyclo [4.5.0]-undec-7-ene (DBU) (3 drops) were added in a Schlenk tube. The mixture was heated at reflux temperature of 170 °C for 48 h under a N_2_ atmosphere. After cooling to room temperature, the precipitate was filtered off and dried in vacuo over P_2_O_5_. The obtained green solid product was purified with column chromatography on silica gel with chloroform/methanol (8:1) as eluent.

*Co(II)Pc* (**5**).Yield: (52%). m.p. = 330 °C. FT-IR (KBr) ν_max_, cm^−1^: 3024 (C-H_arom_); 1387 (C-C); 1270 (C-N); 1607 (C=C); 1480 (C=N); 902 (Co-N). UV/Vis (DMSO, λ_max_ nm (log **ε**)): 340 (5.033), 606 (4.648), 693 (5.243). Calc. for (C_67_H_36_N_8_O_12_Co): calculated (C, 66.83%; H, 3.01%; N, 9.30%); found (C, 66.8%; H, 3.20%; N, 9.4%).

*Zn(II)Pc* (**6**). Elution solvent system: chloroform/methanol (100:3) as eluent. Yield: (66%). m.p. = 330 °C. FT-IR (KBr) ν_max_, cm^−1^: 3020 (C-H_arom_); 1390 (C-C); 1272 (C-N); 1602 (C=C); 1482 (C=N); 903 (Zn-N). UV/Vis (DMSO, λ_max_ nm (log **ε**)): 331 (4.924), 620 (4.653), 690 (5.169). Calc. for (C_67_H_36_N_8_O_12_Zn): calculated (C, 66.48%; H, 2.99%; N, 9.25%); found (C, 66.5%; H, 3.10%; N, 9.3%).

*Cu(II)Pc* (**7**). Yield: (39%). m.p. = 325 °C. FT-IR (KBr) ν_max_, cm^−1^: 3020 (C-H_arom_); 1385 (C-C); 1269 (C-N); 1606 (C=C); 1479 (C=N); 904 (Cu-N). UV/Vis (DMSO, λ_max_ nm (log **ε**)): 345 (4.818), 622 (4.526), 681 (5.074). Calc. for (C_67_H_36_N_8_O_12_Cu): calculated (C, 66.58%; H, 3.00%; N, 9.27%); found (C, 66.5%; H, 3.10%; N, 9.3%).

### 3.5. General Procedure for Preparation of Compounds ***8**–**12***

Ethyl acetoacetate (1 mmol), aldehyde (1 mmol) and urea or urea (1.5 mmol) was heated at reflux for appropriate duration of time. After completion of the reaction as indicated by TLC (hexane/ethyl acetate 8:2), the reaction mixture was brought to room temperature. The remaining solid material was washed with hot ethyl acetate. The filtrate was concentrated and the solid product was recrystallized from ethanol to give the pure product.

*6-Methyl-2-oxo-4-phenyl-1,2,3,4-tetrahydropyrimidine-5–carboxylate* (**8**). Yield: (78%). m.p. = 310 °C. IR (KBr): ν_max_ (cm^−1^) = 3394 (NH); 3217 (NH); 3068 (NH); 1730 (C=O); 1660 (C=O); 1566 (C=C). ^1^H-NMR δ ppm: 1.06 (t, 3H, CH_3_(a)); 2.24 (s, 3H, CH_3_(b)); 3.85 (q, 2H, Hd); 6.74 (s, 1H, H_3_); 6.87 (s, 1H, H_1_); 7.1 (s, 1H, H_6_); ^13^C-NMR δ ppm: 13.5 (CH_3_(b)); 16.6 (CH_3_(a)); 52.5 (C_b_); 60.7 (C_d_); 109.8 (C_5_); 141.6 (C_4_); 158.7 (CO ester); 157.4 (C_2_), 127.4–144.2(C_arom_). Calc. for C_14_H_15_0_3_N_2_ C, 9.82%; H, 88.53%, N, 1.63%; found: C 9.7%; H, 88.4%; N, 1.5%.

*4-(3-Methoxyphenyl)-6-methyl-2-oxo-1,2,3,4-tetrahydropyrimidine-5–carboxylate* (**9**). Yield: (92%). M.p. = 220 °C. IR (KBr): ν_max_ (cm^−1^) = 3361 (NH); 3221 (NH); 1725 (C=O); 1616 (C=O); 1581 (C=C). ^1^H-NMR δ ppm: 1.14 (s,3H, (CH_3_(b)); 1.2 (s, 3H, (CH_3_(c)); 2,19 (s, 3H, CH_3_(a)); 4.50 (s, 2H, H_d_); 6.72 (s, 1H, H_3_); 7.3-8.4 (m, 5H, H_arom_); 5.24 (s, 1H, H_6_). ^13^C-NMR δ ppm: 13.6 (CH_3_); 16.5 (CH_3_(b)); 42.1 (CH_3_(a)); 52.4 (C_6_); 60.6 (C_d_); 109.6 (C_5_); 140.7 (C_4_); 159.6 (CO_ester_); 163,5 (C_1_); 127.5–139.4 (C_arom_), 176,4 (C_3_). Calc. for C_15_H_17_04N_2_ C, 9.35%; H, 89.19%, N, 1.45%; found: C, 9.4%; H, 89.2%; N, 1.5%.

*4-(3-Methylphenyl)-6-methyl-2-oxo-1,2,3,4-tetrahydropyrimidine-5–carboxylate* (**10**). Yield: (96%). M.p. = 315 °C. IR (KBr): ν_max_ (cm^−1^) = 3382 (NH); 3232 (NH); 1720 (C=O); 1652 (C=O); 1570 (C=C). ^1^H-NMR δ ppm: 1.03 (t, 3H, CH_3_(c)); 2.24 (s, 3H, CH_3_(b)); 3.42 (s, 3H, CH_3_(a)); 3.82 (q, 2H, H_d_); 5.85 (s, ^1^H, Hb); 6,85 (dd, 1H, H_6_′); 6.80 (s, 1H, H_2_′); 7.1 (dd, 1H, H_4_′); 7.25 (dd, 1H, H_5_′); 7.6 (s, 1H, H_1_); 8.7 (s, 1H, H_3_). ^13^C-NMR δ ppm: 55.5 (OCH_3_); 16.4 (CH_3_(b)); 42.5 (CH_3_(a)); 52.7 (C_6_); 60,4 (C_d_); 109.5 (C_5_); 140.6 (C_4_); 160.1 (CO_ester_); 156.5 (C_2_); 127.3–139.1 (C_arom_). Calc. for C_15_H_17_0_3_N_2_ C, 9.36%; H, 89.18%, N, 1.45%; found: C, 9.4%; H, 89.2%; N, 1.5%.

*4-(4-Bromophenyl)-6-methyl-2-oxo-1,2,3,4-tetrahydropyrimidine-5–carboxylate* (**11**). Yield: (92%). m.p. = 315 °C. IR (KBr): ν_max_ (cm^−1^) = 3398 (NH); 3219 (NH); 3107 (NH); 1720 (C=O); 1631 (C=O); 1568 (C=C). ^1^H-NMR δ ppm: 1.4 (t, 3H, CH_3_(c)); 2.22 (s, 3H, CH_3_(b)); 2.14 (s, 6H, N(CH_3_)_2_); 3.55 (q, 2H, Hd); 5.85 (s, 1H, H_6_); 8.65 (s, 1H, H_3_); 7.40 (s, 1H, H_1_); 6.62–7.25 (m, 5H, H_arom_). ^13^C-NMR δ ppm: 16.5 (N(CH_3_)_2_); 14.6 (CH_3_(b)); 42.5 (CH_3_(a)); 52.7 (C_6_); 61.1 (C_d_); 109.4 (C_5_); 141.3 (C_4_); 158.8 (CO_ester_); 157.4(C_2_); 125.5–137.3 (Carom). Calc. for C_14_H_14_0_3_N_2_Br C, 9.948%; H, 83.66%, N, 1.65%; found: C, 9.8; H, 83.2, N, 1.7.

*4-(4-Nitrophenyl)-6-methyl-2-oxo-1,2,3,4-tetrahydropyrimidine-5–carboxylate* (**12**). Yield: (85%). m.p. = 315 °C. IR (KBr): ν_max_ (cm^−1^) = 3390.6 (NH); 3218 (NH); 1729 (C=O); 1656 (C=O); 1560.3 (C=C). ^1^H-NMR δ ppm: 1.3 (t, 3H, CH_3_(c)); 2.22 (s, 3H, CH_3_(b)); 3.85 (q, 2H, H_d_); 5.98 (s, 1H, H_6_); 8.69 (s, 1H, H_3_); 7.50 (s, 1H, H_1_); 6.76–7.36 (m,5H, H_arom_). ^13^C-NMR δ ppm: 14.9 (CH_3_(b)); 41.5 (CH_3_(a)); 53.7 (C_6_); 61.3 (C_d_); 108.2 (C_5_); 142.3 (C_4_); 158.6 (CO_ester_); 157. 4 (C_2_); 127.5-139.3 (C_arom_). Calc. for C_14_H_14_O_5_N_3_ C, 10.33%; H, 87.07%; N, 2.58%; found: C, 10.4%; H, 87.2%; N, 2.6%.

### 3.6. Antimicrobial Activities 

Antimicrobial activities of different DHPMs were evaluated by the agar well diffusion method [34] and Minimum inhibitory concentration (MIC) [35,36].

## 4. Conclusions

The present study reports a new method for the preparation of DHPMs was discovered that utilizes a multicomponent coupling reaction catalyzed by MPc, with a rapid and high yielding cyclocondensation to afford the corresponding DHPMs. The use of MPc, was well tolerated by a range of aldehydes. These phthalocyanine complexes were found to be efficient, recyclable heterogeneous catalyst and showed rate enhancements and high yields in this transformation. Hence the use of MPc as a catalyst, for synthesis DHPMs is a precious addition to the available methods. Structures of synthesized compounds have been confirmed by IR, ^1^H-NMR, ^13^C-NMR and mass spectra. The compounds **8**–**12** were investigated for their antimicrobial activities and revealed good activity.

## Figures and Tables

**Table:** 

M	Co	Zn	Cu
Compounds	5	6	7

**Table 1:** Absorption and extinction coefficients of compounds **5**–**7.**

Solvent	Pcs	Bandes (Q), λmax (nm) (log ε)	Bandes (B), λmax (nm) (log ε)
DMF	**5**	615 (4.591); 697 (5.135)	340 (5.033)
	**6**	636 (4.632); 697 (5.064)	343 (4.834)
	**7**	614 (4.436); 675 (4.968)	332 (4.645)
DMSO	**5**	622 (4.462); 708 (5.054)	334 (4.728)
	**6**	631 (4.337); 701 (5.004)	340 (4.526)
	**7**	614 (4.246); 693 (4.885)	351 (4.403)

**Table 2:** Biginelli condensation using different metallophthalocyanines **5**–**7** as catalysts under different reaction conditions.

Entry	Substrate	MPc	Reaction Time (h)	Solvent	Catalyst (mol %)	Yield ^a^ (%)
1	benzaldehyde	**5**	1.5	DMC	2	98
2	benzaldehyde	**6**	3	DMC	2	90
3	benzaldehyde	**7**	2	DMC	2	85
4	3-methoxy benzaldehyde	**5**	2.5	EtOH	2	80
5	3-methoxy benzaldehyde	**5**	3	THF	2	75
6	3-methoxy benzaldehyde	**5**	3.5	CH_3_COCH_3_	5	98
7	3-methoxy benzaldehyde	**5**	1	DMC	10	98
8	3-methoxy benzaldehyde	**5**	4	DMC	-	trace

Reaction conditions: aldehyde (5 mmol), urea (5 mmol), ethyl acetoacetate (5 mmol), solvent (5 mL) and catalyst (2–10 mol %) at refluxing temperature. ^a^ Isolated yields. DMC: dimethylcarbonate.

**Table 3:** Co (II)Pc-catalyzed one pot synthesis of dihydropyrimidinones **8**–**12**.

Substrate	MPc	DHPM	Time (h)	Yield ^a^ (%)
Benzaldehyde	Co(II)Pc	8	3	78
3-Methoxybenzaldehyde	Co(II)Pc	9	4	92
4-Methylbenzaldehyde	Co(II)Pc	10	2	96
4-Bromobenzaldehyde	Co(II)Pc	11	2	92
4-Nitrobenzaldehyde	Co(II)Pc	12	3	85

^a^ Isolated yields.

**Table 4:** Results of reusability of the Co(II)Pc.

Entry	Catalyst (mol)	Reaction Time (h)	Yield ^a^ (%)
1	2	1	98
2	Cycle1	1	90
3	Cycle2	1	85
4	Cycle3	1	95

Cycle 1, 2, 3 indicate the reusability of the catalyst recovered from experiment 1. Reaction condition: Benzaldehyde (5 mmol), ethyl acetoacetate (5 mmol), urea (5 mmol) and catalyst (2 mol %) in refluxing DMC. ^a^ Isolated yields.

**Table 5:** Determination of the Minimum Inhibitory Concentrations (MICs) expressed in mg/mL.

Indicator Microorganism	Compounds	MIC (mg/mL)
*Micrococcus luteus* *LB 14110*	8	10
	9	1.25
	10	4
	11	1.5
	12	8
*Staphylococcus aureus* *ATCC 6538*	8	5
	9	2.5
	10	5
	11	4
	12	-
*Listeria monocytogenes* *ATCC 19117*	8	5
	9	0.625
	10	-
	11	5
	12	3.5
*Salmonella Typhimurium* *ATCC 14028*	8	10
	9	5
	10	-
	11	5
	12	8
